# Open Competitive Examination of M. D.’s

**Published:** 1891-06

**Authors:** 


### OPEN COMPETITIVE EXAMINATION OF M. D.'S.

An open competitive examination of candidates for Junior Assistants and Female Physicians, in the State Hospitals for the Insane, will be held by the State Civil Service Commission at the Capitol in Albany, June 11th. Candidates must be residents of the State, and must have had one year's experience in a hospital, or three years' experience in the general practice of medicine.

---

THE CHICAGO WOMEN'S NEWS has inaugurated a Legal Department of Law, which is presided over by Attorney Mary A. Ahrens. This is quite a novel feature in this class of journalism, and as "Law is the perfection of reason," we shall look for some happy illustrations of it in the Law columns of the News.

FOR NERVOUS PROSTRATION.—Dr. W. Graves, Northfield, Minn, says: "I have used it in cases of Nervous Prostration, and also in combination with other remedies; in Indigestion, it has proved as satisfactory as could be expected."

GETTING MARRIED AND KEEPING MARRIED, is the title of No. 18, of the "Human Nature Library," put forth by the Fowler & Wells Co., Publishers, 775 Broadway. The author is one who has had experience in both branches of his subject—he married and keeps married, and his methods are doubtless worthy of imitation. As for ourself, it comes too late.

